# Rapid and Reliable Detection of Nonsyndromic Hearing Loss Mutations by Multicolor Melting Curve Analysis

**DOI:** 10.1038/srep42894

**Published:** 2017-02-22

**Authors:** Xudong Wang, Yongjun Hong, Peihong Cai, Ning Tang, Ying Chen, Tizhen Yan, Yinghua Liu, Qiuying Huang, Qingge Li

**Affiliations:** 1 State Key Laboratory of Cellular Stress Biology, State Key Laboratory of Molecular Vaccinology and Molecular Diagnostics, Engineering Research Centre of Molecular Diagnostics, Ministry of Education, School of Life Sciences, Xiamen University, Xiamen, Fujian 361102, China; 2 Department of Otorhinolaryngology, Zhongshan Hospital of Xiamen, Xiamen University, Xiamen, Fujian, 361004, China; 3 Department of Medical Genetics, Liuzhou Key Laboratory of Birth Defects Prevention and Control, Liuzhou Maternal and Child Health Hospital, Liuzhou, Guangxi 545001, China; 4 Nanjing Medical University Affiliated Wuxi Maternity and Child Health Care Hospital, Jiangsu 215002, China; 5 Department of Neonatology, Central Lab Suzhou Hospital Affiliated to Nanjing Medical University, Suzhou, Jiangsu 215002, China

## Abstract

Hearing loss is a common birth defect worldwide. The *GJB2, SLC26A4, MT-RNR1* and *MT-TS1* genes have been reported as major pathogenic genes in nonsyndromic hearing loss. Early genetic screening is recommended to minimize the incidence of hearing loss. We hereby described a multicolor melting curve analysis (MMCA)-based assay for simultaneous detection of 12 prevalent nonsyndromic hearing loss-related mutations. The three-reaction assay could process 30 samples within 2.5 h in a single run on a 96-well thermocycler. Allelic types of each mutation could be reproducibly obtained from 10 pg ~100 ng genomic DNA per reaction. For the mitochondrial mutations, 10% ~ 20% heteroplasmic mutations could be detected. A comparison study using 501 clinical samples showed that the MMCA assay had 100% concordance with both SNaPshot minisequencing and Sanger sequencing. We concluded that the MMCA assay is a rapid, convenient and cost-effective method for detecting the common mutations, and can be expectedly a reliable tool in preliminary screening of nonsyndromic hearing loss in the Chinese Han population.

Hearing loss (HL) is one of the most prevalent birth defects in humans, affecting about 1 ~ 2 out of every 1000 infants^1^. Among them, about 50% is attributed to the genetic factors and approximately 70% are non-syndromic hearing loss (NSHL, only the symptom of hearing loss). To date, more than 100 different genes have been identified for NSHL in human involving autosomal dominant, autosomal recessive, X-linked and maternal inheritance patterns (http://hereditaryhearingloss.org). Currently, large epidemiological studies have showed that *GJB2, MT-RNR1*, and *SLC26A4* genes are the most common causes in Chinese NSHL population^2^,^3^,^4^. Mutations in connexin 26 gene (*GJB2*) are the most common cause of NSHL, accounting for nearly half of patients. To date, more than 100 mutations in *GJB2* have been identified (http://davinci.crg.es/deafness/index.php) and the mutation spectrum varies among different ethnic backgrounds. The mutations of c.35delG, c.167delT and c.235delC are the most frequent mutations in Caucasian^5^, Ashkenazi Jews^6^, and Asian populations^7^,^8^, respectively. The solute carrier family 26, member 4 (*SLC26A4*), encoding a transmembrane protein, is considered as the second most common responsible gene for NSHL and Pendred syndrome. So far, more than 160 mutations have been identified in *SLC26A4* gene (http://deafnessvariationdatabase.org/letter/s), but the mutation of c.919-2A > G is the most common causative mutation in Asian population^9^,^10^. Additionally, the mutations in *MT-RNR1* gene and *MT-TS1* gene, encoding 12S rRNA and the transfer RNA for serine, respectively, are major contributors to NSHL or aminoglycoside-induced deafness, which may have an effect of increasing susceptibility to aminoglycoside ototoxicity^11^,^12^. These mutations collectively form the basis for neonatal screening of HHL in many countries.

Successful implementation of a neonatal screening program or genetic testing is largely dependent on the pattern of high frequency mutations in populations and the detection methods used. So far, a variety of molecular methods have been developed for detecting NSHL-related mutations. These included allele specific oligonucleotide analysis^5^, denaturing high-performance liquid chromatography (DHPLC)^13^, high resolution melting analysis (HRM)^14^, SNaPshot minisequencing technology^15^, microarray-based approach^16^, APEX array technology^17^, ARMS-PCR analysis^18^, Invader assay^19^, multiplex allele-specific PCR-based universal array (ASPUA)^20^, mass spectroscopy^21^, SNPscan assay^22^, MassARRAY iPLEX^®^ technology^23^, massively parallel sequencing^24^ and Sanger sequencing. So far, the microarray-based approach is the only regulatory agency approved assay in China and is also the most widely used method. However, its implementation in clinical settings is often hindered by the requirement of high end instrument, complex and lengthy operation, cost-ineffectiveness and small coverage of mutations.

Multicolor melting curve analysis (MMCA) is a new mutation detection method based-on real-time PCR and it featured by detecting multiple mutations in a single reaction. The accuracy, ease-of-use, rapidity and cost-effectiveness have allowed the successful use of MMCA for β- and α-thalassmia screening^25^,^26^,^27^. In this study, we attempted to apply MMCA to establish a screening protocol for the 12 common mutations in Chinese population. We systematically evaluated both analytical and clinical performances of the MMCA assay. Comparison studies between the MMCA assay and the SNaPshot assay as well as Sanger sequencing were performed using clinical samples of varied sources.

## Results

### Design and establishment of the MMCA assay

**Figure 1:**
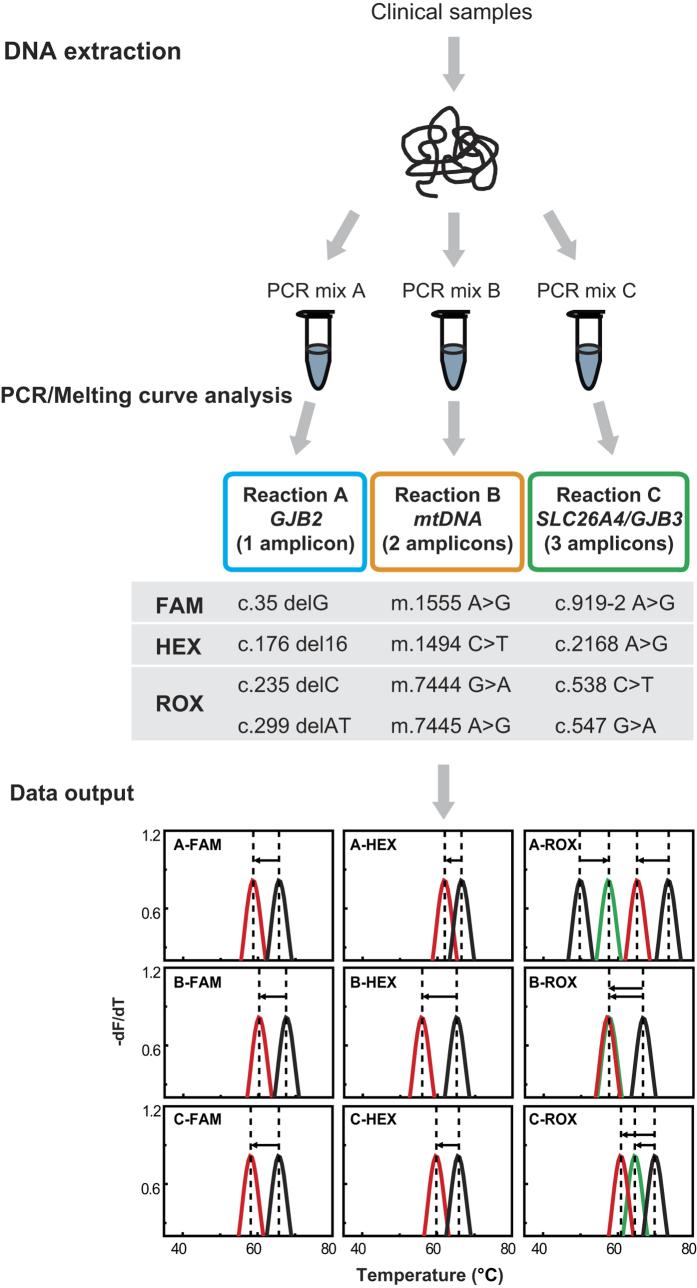
The workflow of MMCA assay for the detection of nonsyndromic hearing loss-associated mutations. Genomic DNA was extracted from the clinical samples and then added into three PCR reactions (each reaction contains the primers and the detection probes labeled three different labeled fluorophores of FAM, HEX or ROX), and subsequently PCR amplification and melting curve analysis were performed on CFX96^TM^ real-time system. The graphic information containing *T*_*m*_ values was automatically produced by the software. The genotypes of *GJB2, MT-RNR1, MT-TS1, SLC26A4* and *GJB3* could be confirmed by the values of *T*_*m*_ and Δ*T*_*m*_. Black lines and colored lines represent for the melting peaks of the wild-type and their corresponding mutations types, respectively.

**Figure 2:**
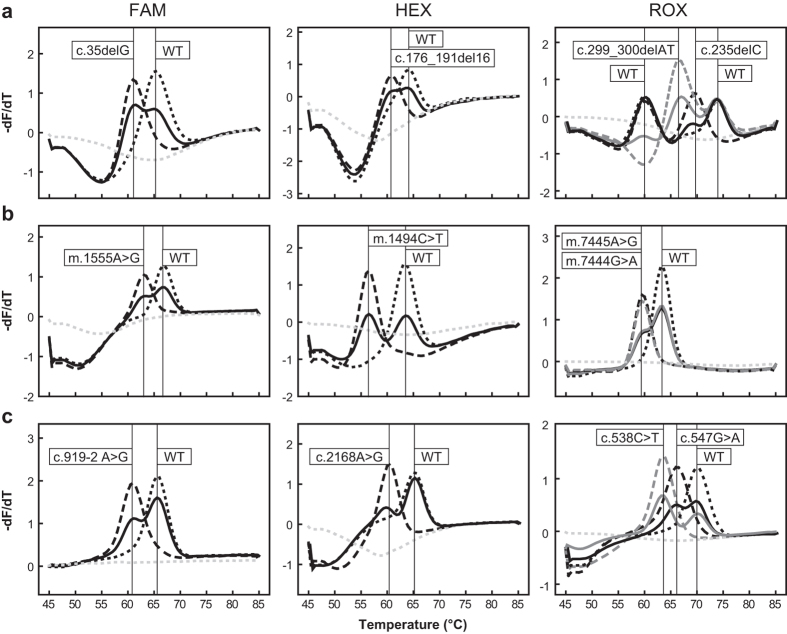
Genotyping results of nonsyndromic hearing loss-associated mutations by the MMCA assay. Melting curves and corresponding genotypes (**box**) of the 12 mutations and wild-type plasmid DNA templates are given according to the detection channels, shown by the corresponding fluorophores. (**a**–**c**) Represent the three reactions, respectively. Gray dotted lines indicate no-template control.

The workflow of the MMCA assay is shown in Fig. 1. Extracted DNA from each sample is added to the PCR tubes, PCR amplification and the melting curve analysis are performed as programmed, and when completed, graphic output with *T*_*m*_ values is automatically generated. For each sample, MMCA assay had three reactions to detect the 12 common mutations, which represent a high proportion of known pathogenic variants for hereditary hearing loss in the Chinese NSHL population from large epidemiological data. Reaction A covers four *GJB2* mutations: c.35delG, c.176_191del16, c.235delC and c.299_300delAT. Reaction B includes four mitochondrial mutations: m.1494C > T, m.1555A > G, m.7444G > A, and m.7445A > G. Reaction C detects two *SLC26A4* mutations: c.919-2A > G and c.2168A > G), and two *GJB3* mutations: c.538C > T and c.547G > A. Except the two mitochondrial mutations (m.7444G > A and m.7445A > G), which are functionally identical (aminoglycoside sensitivity) and are not differentiated from each other, all other 10 mutations are all individually identified by their *T*_*m*_ values, labeling fluorophores and the reaction names (Fig. 2).

### Analytical studies

The reproducibility study showed that the *T*_*m*_ values had the three times standard deviation (3SD) ranged from 0.30 to 0.87, and all the 12 mutations (with the exception of the undifferentiated m.7444G > A and m.7445A > G) could be resolved with *T*_*m*_ difference larger than 2.5 °C, which is much larger than the 3SD values (Table 1).

The analytical sensitivity study showed that 10 pg gDNA per reaction could be repeatedly detected (see Figs S1, S2 and S3 in the supplemental material). The detectable amounts of gDNA ranged from 10 pg to 100 ng gDNA per reaction.

**Figure 3:**
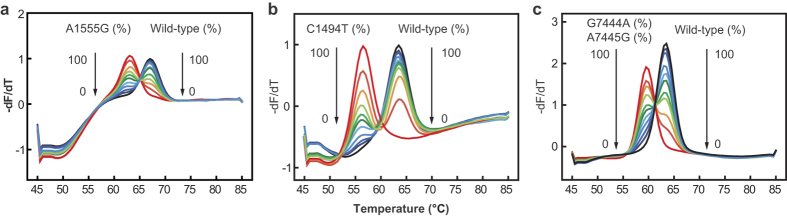
Melting curves for different percentages of mtDNA mutations. Melting curves were from mimic mitochondrial plasmid DNA samples with different percentages (from 0%, 10%, 20%, 30%, 40%, 50%, 60%, 70%, 80%, 90%, to 100%) of A1555G (**a**), C1494T (**b**), and G7444A/A7445G mutation (**c**). Both wild-type and mutant-type templates were plasmid DNAs and the concentration was set at 1** ×** 10^4^ copies per reaction.

The heteroplasmic study showed that DNA sample containing 10% or 20% mutant DNA when at 2 × 10^4^ copies/μL could be distinctly detected from the wild-type (Fig. 3).

### Clinical evaluation

The MMCA assay results of 293 gDNA samples were listed in Table 2. In total, 16 genotypes were detected. Among them, the carrier frequencies of mutations in *GJB2, SLCA26A4*, mtDNA and *GJB3* were 9.90% (29/293), 6.14% (18/293), 12.29% (36/293) and 0.68% (2/293), respectively. By referring to the SNaPshot assay results, a complete concordance between the two methods was obtained.

To investigate the potential of MMCA assay for screening use, 208 sporadic hearing impaired patients referred to Zhongshan Hospital of Xiamen were recruited and analyzed using their salivary gDNA samples. The results showed that 63 (30.3%, 63/208) patients harbored one or more causative mutation, of which 37 (17.8%, 37/208) patients had two mutant alleles and 26 (12.5%, 26/208) patients had a single mutant allele (see Table S3 for further details). In total, 7 mutations and 14 genotypes were obtained from this cohort. A parallel Sanger sequencing analysis again gave fully consistent results.

We then calculated the turnaround time and cost of the MMCA assay and compared them with Sanger sequencing. One round of MMCA assay that processes 30 samples required 10 min for DNA adding, 2.5 h for PCR and melting analysis. From the graphic information given by the machines, the genotypic results could be obtained artificially within less than 5 min. Thus the entire assay could be finished within 3 h when DNA and PCR master mix are ready for use. In one working day, two rounds of MMCA assay could be finished and 60 (2 × 30) unknown samples could be analyzed. By comparison, Sanger sequencing was carried out by a commercial service that can be finished overnight when PCR products are received. Because each sample needs to run 6 PCRs to prepare the sequencing template, and 60 samples require 360 PCRs in total. Thus the time needed for sequencing is doubled compared with MMCA even at the PCR stage without consideration the custom service that needs additional 2 days. The material cost of MMCA assay for each sample was estimated to be 5 USD. The automatic DNA extraction cost was less than 2.5 USD for each sample according to the list price. Thus, the overall material cost for each sample could be no more than 10 USD when other consumables were considered. For Sanger sequencing, the service cost is 3 USD for each run according to the list price, thus one sample needs 18 USD. The overall cost for Sanger sequencing including DNA extraction, PCR, and service cost will be more than 20 USD for one sample. Taken together, either the turnaround time or the material cost spent on one sample, the MMCA assay is less than one-half those of Sanger sequencing.

## Discussion

HL is the most common neurosensory disorder in humans. Although universal newborn hearing screening (UNHS) plays an important role in early identification of NSHL, it could not detect the subjects with mild NSHL and those at risk for late-onset NSHL that could be exactly detected by genetic screening^28^. In this study, a new MMCA assay was successfully established to detect the 12 hot-spot mutations in Chinese NSHL population. The analytical results showed that each mutation could be unambiguously genotyped in a wide range of gDNA (from 10 pg to 100 ng gDNA per reaction), and 10% or 20% mutant DNA could be distinctly detected in the heteroplasmic samples. The clinical studies of 293 gDNA samples as well as 208 patients showed that the MMCA assay were as accurate as Sanger sequencing and SNaPshot assay. Cost and turnaround analysis further displayed that it was more effective than Sanger sequencing in both.

The above merits combined with the closed-tube feature render MMCA assay an alternative approach to existing strategies for NSHL screening. Large scale neonatal screening programs for NSHL have been increasingly implemented in China since the introduction a regulatory agency approved DNA chip assay, which detects 9 mutations in *GJB2, SLC26A4*, mitochondria, and *GJB3*^20^. Other methods such as SNaPshot^15^ and MS-based assay^21^ are also recommended for this use. However, all these methods involve lengthy and complex post-PCR manipulations, which not only increase the workload of the operators but also invite false results incurred by PCR products contaminations. Consequently, these methods often require well-trained personnel and dedicated facility, hindering the wide spread of the HHL screening program. By contrast, as a closed-tube system, MMCA assay was easy to use, had negligible chance to get contaminated by PCR products, and thus could be easily operated in neonatal screening centers of different levels.

MMCA assay can be run in a real-time PCR thermocycler, an inexpensive instrument often found in a standard molecular biology laboratory. Thus, investment of high-end instruments such as a sequencer or a MS spectrometer is unnecessary for commencement of HL screening program. Our previous study showed that MMCA could be used in all mainstream real-time PCR machines without any program change^25^. The program used in the Bio-Rad CFX 96 machine could be easily adapted to other models. Currently, we have already tested the assay in five different real-time PCR instruments, including Rotor-Gene^TM^ 6000 real-time rotary analyzer (Corbett Research, Mortlake, Australia), CFX 96^TM^ real-time PCR detection system (Bio-Rad, Hercules, CA), ABI 7500 (Life Technologies, Carlsbad, CA), LightCycler^®^ 480 (Roche Applied Sciences, Indianapolis, IN) and SLAN-96S real-time PCR system (Hongshi Medical Technology Co., Ltd, Shanghai, China), and all gave similar results. Also, the software for automatic genotype interpretation had been developed for an inexpensive China-made real-time thermocycler, which should facilitate domestic implementation of the MMCA assay.

Another advantage of the MMCA assay is its wide range of template concentration, and in particular, very low amount (10 pg gDNA per reaction) could be used. This might allow the use of a variety of sources of clinical samples for HHL screening, including peripheral blood, newborn dried bloodspot, amniotic fluid, etc. In this study, 293 gDNA samples were extracted from bloodspot cards routinely used for neonatal screening program, and 208 samples were obtained from the patients’ saliva. The compatibility with different resources of samples would facilitate the use of MMCA assay in different settings. In particular, saliva sampling is simple, non-invasive, and requires no trainings compared with sampling of blood. It would also lower the cost and the infection risk as well. Salivary DNA had been successfully used for genotyping analysis^29^,^30^. Implementation of saliva sampling should be especially interesting for large-scale neonatal screening program of HHL.

One limit of MMCA assay might be the restricted number of mutations included. HHL is a complex disease, involving 154 genes and some of which have hundreds of causative mutations (http://hereditaryhearingloss.org/main.aspx?c=.HHH&n=86596). The prevalent frequency of the mutations among HHL varies with ethnic groups and geographic locations^31^. So far, a database of Chinese mutation spectrum of HHL remains to be available. The 12 mutations chosen from the five genes, i.e., *GJB2, GJB3, MT-RNR1, MT-TS1* and *SLC26A4*, represent only those frequently found according to current epidemiological studies^32^. As shown in the results for the 208 salivary samples collected from the sporadic moderate-to-profound hearing loss patients, *GJB2* and *SLC26A4* contributed most mutations, of which, 235delC in *GJB2* (43/95, 45.3%) and c.919-2A > G in *SLC26A4* (33/95, 34.7%) appeared to be the most common mutations. This distribution of mutation was consistent with other regions in China^2^,^33^, showing the applicability of these mutations chosen for HHL screening across this country. Studies on the Chinese mutations spectrum of HHL are now in progress assisted with next-generation sequencing, and a more comprehensive MMCA assay could be expected in the near future.

In summary, we described a closed-tube MMCA assay for HHL screening. The assay is rapid, easy-to-use, cost-effective, and is as accurate as Sanger sequencing. While the included mutations are limited at current stage, its usefulness was proved in the clinical studies. It could be expectedly used as an efficient first-line screening tool on a large-population base in China.

## Materials and Methods

The methods were carried out in accordance with the approved guidelines. The research was approved by the Research Ethics Committee of Liuzhou Maternity and Child Health Care Hospital, Nanjing Medical University Affiliated Suzhou Hospital and Zhongshan Hospital Affiliated to Xiamen University and followed the approved ethical standards of the declaration of Helsinki. Moreover, the informed consent for this study was obtained from all the subjects or their guardians.

### DNA templates and Clinical samples

For the optimization and the standardization of the MMCA assay, 12 genomic DNA (gDNA) samples with known genotypes, i.e., 5 wild-type, 1c.35delG/N, 1c.235delC/N, 1c.299_300delAT/N, 1c.547G > A/N, 1c.919-2A > G/N, 1c.2168A > G/N and 1 m.1555A > G, provided by Liuzhou Maternal and Child Health Care Hospital were used. Artificial plasmid DNA templates containing individual mutations of c.35delG, c.176_191del16, c.235delC, c.299_300delAT of *GJB2*, c.919-2A > G, c.2168A > G of *SLC26A4*, c.538C > T, c.547G > A of *GJB3*, m.1494C > T, m.1555A > G of *MT-RNR1* and m.7444G > A, m.7445A > G of *MT-TS1* gene were prepared respectively by PCR-mediated *in vitro* mutagenesis and the presence of the mutations were confirmed by bi-directional Sanger sequencing.

For clinical evaluation, 293 gDNA samples were collected from Liuzhou Maternity and Child Health Care Hospital and Nanjing Medical University Affiliated Suzhou Hospital. The gDNA was extracted from bloodspot card using Lab Aid 96 automatic DNA extraction system (Zeesan Biotech, Xiamen, China). The concentration of each gDNA sample was determined by ND-1000 UV-VIS spectrophotometer (NanoDrop Technologies Inc, Wilmington, USA). All these samples were previously genotyped by the SNaPshot assay, which can detect 13 causative mutations, i.e., c.35delG, c.176_191del16, c.235delC, c.299_300delAT of *GJB2*, c.919-2A > G, c.2168A > G, c.1174A > T, c.1229C > T, and c.2027T > A of *SLC26A4*, m.1494C > T, m.1555A > G of *MT-RNR1* and m.7444G > A, m.7445A > G of *MT-TS1* gene. And all the gDNA samples were re-numbered and their identities were blinded to the individuals who performed the testing. Additionally, salivary samples of 208 sporadic hearing loss patients from Zhongshan Hospital of Xiamen were recruited. None of them had any syndromic findings and all patients showed pre-lingual deafness and had moderate to profound hearing loss. gDNA was extracted from the salivary samples by using Lab-Aid 820 nucleic acid extraction system (Zeesan Biotech, Xiamen, China) according to the manufacturer’s instructions. The purity and concentration of gDNA was determined by ND-1000 UV-Vis spectrophotometer and store in −20 °C before use. All clinical samples were obtained with written informed consent and in accordance with the guideline approved by Liuzhou Maternity and Child Health Care Hospital Ethics Committee, Nanjing Medical University Affiliated Suzhou Hospital Ethics Committee and Zhongshan Hospital Affiliated to Xiamen University Ethics Committee.

### Primers and Probes

DNA sequences of *GJB2, GJB3, MT-RNR1, MT-TS1* and *SLC26A4* were obtained from the NCBI website (http://www.ncbi.nlm.nih.gov/). To detect the 12 hot-spot mutations of HHL in Chinese population, six pairs of primers and ten dual-labeled self-quenched probes (see Table S1 in the supplemental material) were designed using Primer5.0 (PREMINER Biosoft International, Palo, CA), Tm Utility v1.3 (Idaho Technologies Inc., Salt Lake City, UT), and Oligo 6.0 (AVG Technologies Inc., Chelmsford, MA). All the primers and probes were synthesized and purified by Sangon (Shanghai, China).

### PCR and Melting Curve Analysis

The three-reaction MMCA assay was performed on CFX96^TM^ real-time system (BIO-RAD, Hercules, USA). Each 25 μL PCR mixture contained 1 × PCR buffer (10 mM Tris-HCl, pH 8.0, 50 mM NaCl, 50% (V/V) glycerol), 0.2 mM dNTPs, 3.0 mM MgCl_2_, 1.0 U Taq HS DNA polymerase (TaKaRa, Dalian, China), 0.06~0.11 μM limiting primers, 0.6–1.1 μM excess primers, 0.1–0.6 μM probes, and 5 μL DNA template. The detailed concentrations of primers and probes are shown in Table S1. The cycling program was as follows: 95 °C for 10 min; 10 cycles of denaturation at 95 °C for 15 s, annealing at 65 °C for 15 s (−1 °C/cycle), and extension at 76 °C for 20 s; 45 cycles of denaturation at 95 °C for 15 s, annealing at 55 °C for 15 s, and extension at 76 °C for 20 s; 95 °C for 1 min; hybridization at 37 °C for 3 min; an incremental temperature rise from 45 °C to 85 °C at a ramp rate of 0.5 °C/step with a 5-s stop between each step. Fluorescence from the FAM, HEX and ROX channels was collected at the annealing step during the second 45 cycles and at each step during the last melting stage. Melting curves were obtained by plotting the negative derivative of fluorescence with respect to temperature versus temperature (−*dF*/*dT*) and the *T*_*m*_ values were obtained automatically from the melting curves through the CFX Manager 3.0 software.

### Analytical study

To study the reproducibility of *T*_*m*_ measurement, 25 plasmid DNA samples (including 1 wild-type, 12 heterozygotes and 12 homozygotes of each mutation) of different mutant genotypes were analyzed by two technicians and each sample analyzed in five replicates. The *T*_*m*_ value of each genotype and the *T*_*m*_ difference (Δ*T*_*m*_) between the wild-type and mutant samples were measured.

To study the analytical sensitivity of the MMCA assay, four gDNA samples from unaffected subjects’ blood were serially 10-fold diluted with DNA solution, yielding gDNA concentrations ranged from 20 ng/μL to 2 pg/μL. Each concentration was analyzed in triplicate.

To study the ability of detection heteroplasmic mitochondrial mutations of MMCA assay, six plasmids (2 × 10^4^ copies/μL) including two wild-types and four mutants of 1494C > T, 1555A > G, 7444G > A, and 7445A > G, respectively, were used to generate mixed DNA containing 0%, 10%, 20%, 30%, 40%, 50%, 60%, 70%, 80%, 90%, and 100% mutant DNA, respectively, and each was analyzed in triplicate.

### Clinical study

To validate the clinical performance of the MMCA assay, 293 blinded gDNA samples were firstly analyzed for the HHL mutations in a blind format. The genotypes detected by the MMCA assay were compared with their genotypes previously determined by SNaPshot minisequencing. Those samples that displayed discordant genotypes were subject to Sanger sequencing.

To simulate a screening scenario, 208 salivary samples collected from Zhongshan Hospital of Xiamen were subject to MMCA assay and Sanger sequencing in parallel (the primers used for sequencing are listed in Table S2 in the supplemental material). The turnaround time and the cost were estimated for both.

## Additional Information

**How to cite this article:** Wang, X. *et al*. Rapid and Reliable Detection of Nonsyndromic Hearing Loss Mutations by Multicolor Melting Curve Analysis. *Sci. Rep.* **7**, 42894; doi: 10.1038/srep42894 (2017).

**Publisher's note:** Springer Nature remains neutral with regard to jurisdictional claims in published maps and institutional affiliations.

## Supplementary Material

Supplementary Information

## Figures and Tables

**Table 1:** Calibrated *T*_*m*_ values of 12 mutations detected using the MMCA assay.

	Color	Genotype^*^	*T*_*m*_ ± 3 SD	Δ*T*_*m*_ ± 3 SD^b^
Reaction A	FAM	WT	65.4 ± 0.51	—
		c.35delG	60.9 ± 0.60	4.5 ± 0.60
	HEX	WT	64.0 ± 0.30	—
		c.176_191del16	61.5 ± 0.48	2.5 ± 0.52
	ROX	WT	59.9 ± 0.60	—
		c.299_300delAT	66.4 ± 0.58	6.5 ± 0.30
	ROX	WT	73.8 ± 0.72	—
		c.235delC	69.3 ± 0.82	4.5 ± 0.39
Reaction B	FAM	WT	66.8 ± 0.87	—
		m.1555A > G	63.3 ± 0.60	3.5 ± 0.64
	HEX	WT	63.5 ± 0.33	—
		m.1494C > T	56.5 ± 0.42	7.0 ± 0.42
	ROX	WT	63.3 ± 0.81	—
		m.7444G > A	59.5 ± 0.30	3.8 ± 0.66
		m.7445A > G	59.5 ± 0.31	3.8 ± 0.60
Reaction C	FAM	WT	65.6 ± 0.74	—
		c.919–2A > G	61.4 ± 0.66	4.2 ± 0.84
	HEX	WT	65.0 ± 0.30	—
		c.2168A > G	61.0 ± 0.54	4.0 ± 0.33
	ROX	WT	69.6 ± 0.51	—
		c.538C > T	63.1 ± 0.30	6.5 ± 0.45
		c.547G > A	65.6 ± 0.33	4.0 ± 0.60

^a^WT, wild-type.

^b^Δ*T*_*m*_ = *T*_*m* (wild-type)_ − *T*_*m* (mutant)_; —, not applicable.

**Table 2:** Genotypes of 293 clinical samples analyzed using the MMCA assay in a blind study.

Genotypes^a^					No. samples^b^		
*GJB2*	*GJB3*	*SLC26A4*	*MT-RNR1*	*MT-TS1*	MMCA	SNaPshot	Sequencing
c.35delG/N	WT	WT	WT	WT	2	2	ND
c.176_191del16/N	WT	WT	WT	WT	3	3	ND
c. 235delC/N	WT	WT	WT	WT	15	15	ND
c.235delC/c.235delC	WT	WT	WT	WT	2	2	ND
c.235delC/N	WT	c.919-2A > G/N	WT	WT	1	1	ND
c.35delG/c.235delC	WT	WT	WT	WT	1	1	ND
c. 299_300delAT/N	WT	WT	WT	WT	5	5	ND
WT	WT	c.919-2A > G/N	WT	WT	13	13	ND
WT	WT	c.919-2A > G/c.919-2A > G	WT	WT	2	2	ND
WT	WT	c.2168A > G/N	WT	WT	2	2	ND
WT	WT	WT	m.1494C > T hom	WT	2	2	ND
WT	WT	WT	m.1555A > G het	WT	1	1	ND
WT	WT	WT	m.1555A > G hom	WT	5	5	ND
WT	WT	WT	WT	m.7444G > A hom	28	28	ND
WT	c.547G > A/N	WT	WT	WT	2	NT	2
WT	WT	WT	WT	WT	209	209	ND
Total					293	291	2

^**a**^WT, wild-type; het, heteroplasmic for the m.1555A > G mutation; hom, homoplasmic for the m.1555A > G, m.1494C > T and m.7444G > A mutation. ^**b**^NT, not tested; ND, not done.
