# Supplementary material for: Rapid and Reliable Detection of Nonsyndromic Hearing Loss Mutations by Multicolor Melting Curve Analysis
Source: Sci Rep. 2017 Feb 22;7:42894. doi: 10.1038/srep42894 (PMC5320477; doi:10.1038/srep42894)
Supplement: Supplementary Information [file srep42894-s1.pdf]

Rapid and Reliable Detection of Nonsyndromic Hearing Loss Mutations by Multicolor Melting Curve Analysis

Xudong Wang [1], Yongjun Hong [2], Peihong Cai [1], Ning Tang [3], Ying Chen [4], Tizhen Yan [3], Yinghua Liu [5], Qiuying Huang [1,*], Qingge Li [1,*]

[1] State Key Laboratory of Cellular Stress Biology, State Key Laboratory of Molecular Vaccinology and Molecular Diagnostics, Engineering Research Centre of Molecular Diagnostics, Ministry of Education, School of Life Sciences, Xiamen University, Xiamen, Fujian 361102, China.

[2] Department of Otorhinolaryngology, Zhongshan Hospital of Xiamen, Xiamen University, Xiamen, Fujian, 361004, China.

[3] Department of Medical Genetics, Liuzhou Key Laboratory of Birth Defects Prevention and Control, Liuzhou Maternal and Child Health Hospital, Liuzhou, Guangxi 545001, China.

[4] Nanjing Medical University Affiliated Wuxi Maternity and Child Health Care Hospital, Jiangsu 215002, China.

[5] Department of Neonatology, Central Lab Suzhou Hospital Affiliated to Nanjing Medical University, Suzhou, Jiangsu 215002, China.

* Correspondance: qgli@xmu.edu.cn, hqying@xmu.edu.cn.

Supplementary Figure S1

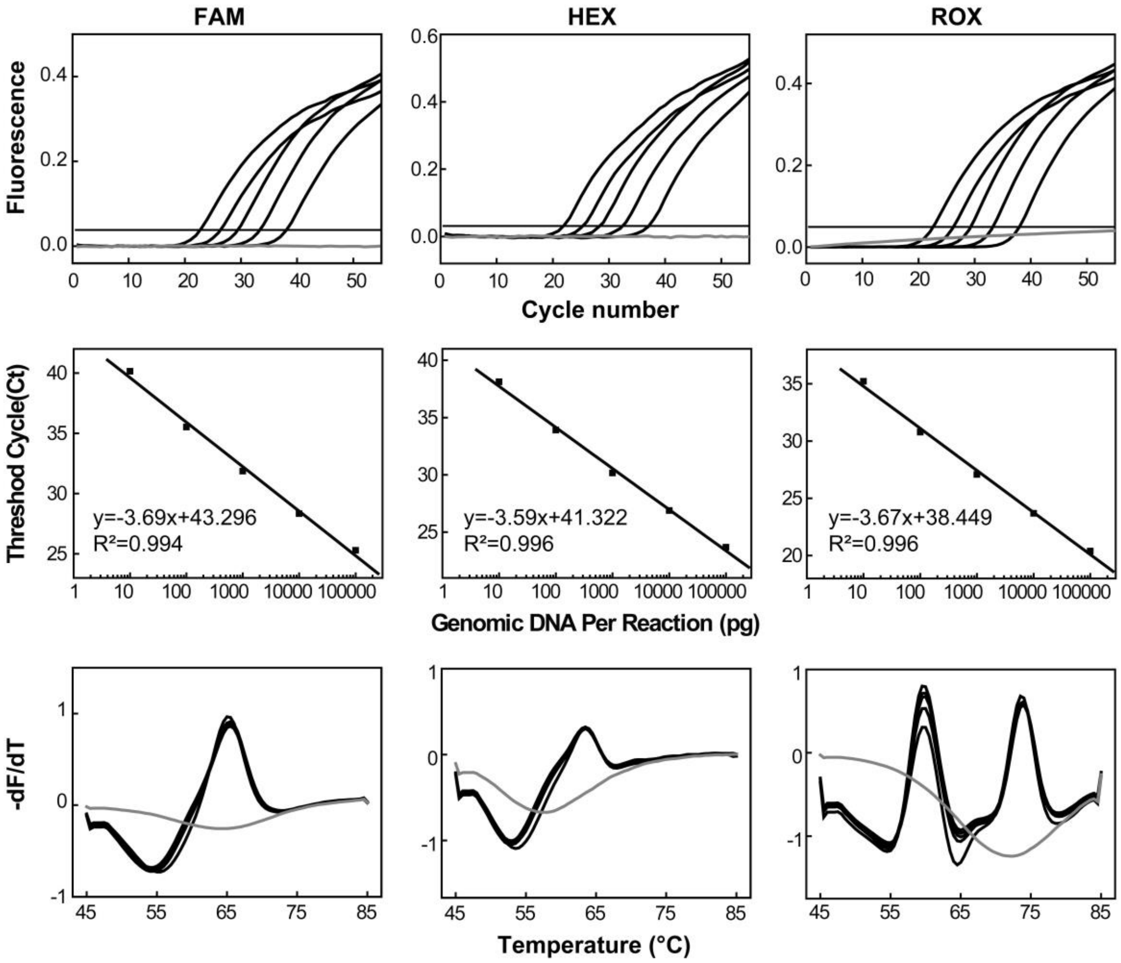

Figure S1. Analytical Sensitivity of Reaction A (*GJB2*) by the MMCA assay. Tenfold serial dilutions of wild-type genomic DNA template ranging from 100 ng to 10 pg (from left to right) in a 25 μL PCR-reaction were performed in reaction A with three detection channels (**FAM, HEX and ROX**). **Top panel**: the PCR amplification curves in different template concentrations. **Middle panel:** Linear relationship between the number of threshold cycle values (Ct) and the logarithm of the concentration of gDNA templates. **Bottom panel:** Melting curves in different DNA templates concentration. And DNA solution was used as the no-template control (**gray lines**).

Supplementary Figure S2

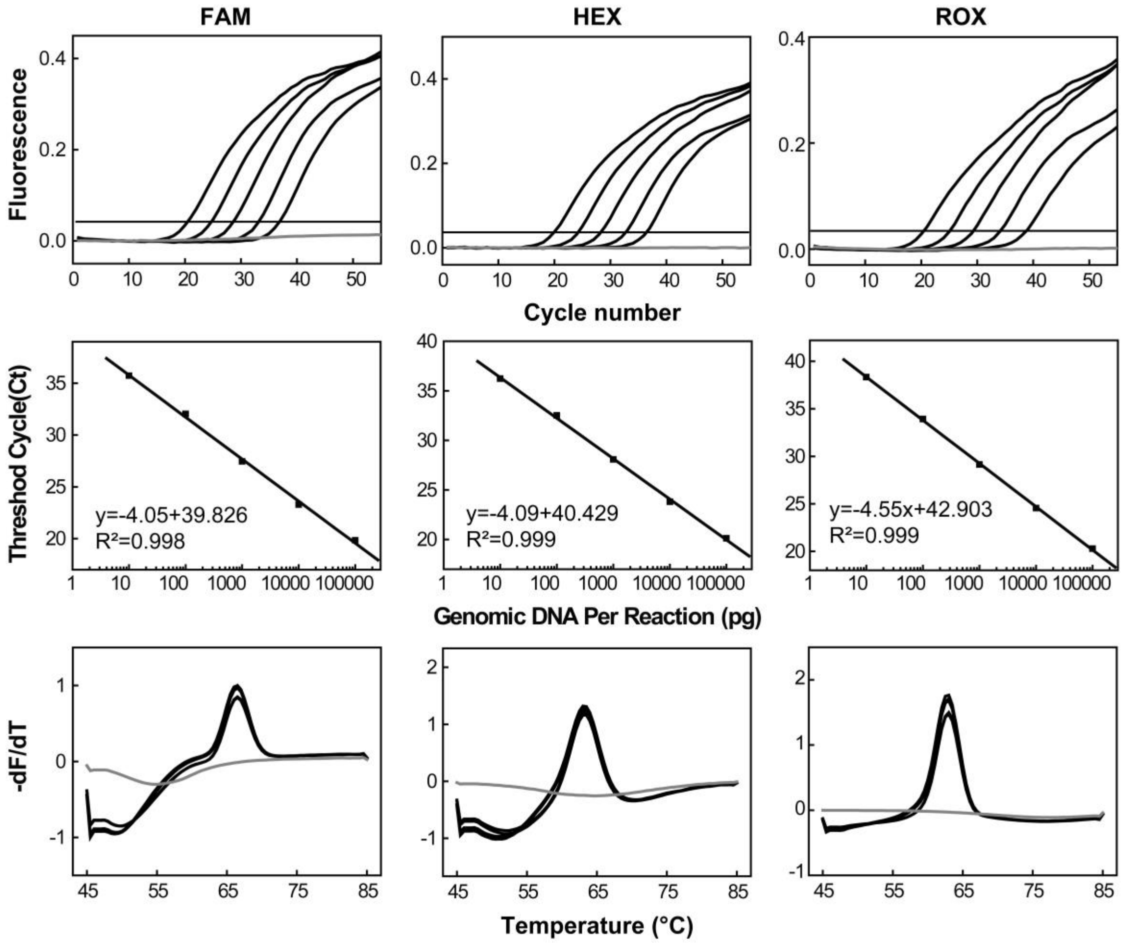

Figure S2. Analytical Sensitivity of Reaction B (mtDNA) by the MMCA assay.

Tenfold serial dilutions of wild-type genomic DNA template ranging from 100 ng to 10 pg (from left to right) in a 25 μL PCR-reaction were performed in reaction A with three detection channels (**FAM, HEX and ROX**). **Top panel**: the PCR amplification curves in different template concentrations. **Middle panel:** Linear relationship between the number of threshold cycle values (Ct) and the logarithm of the concentration of gDNA templates. **Bottom panel:** Melting curves in different DNA templates concentration. And DNA solution was used as the no-template control (**gray lines**).

Supplementary Figure S3

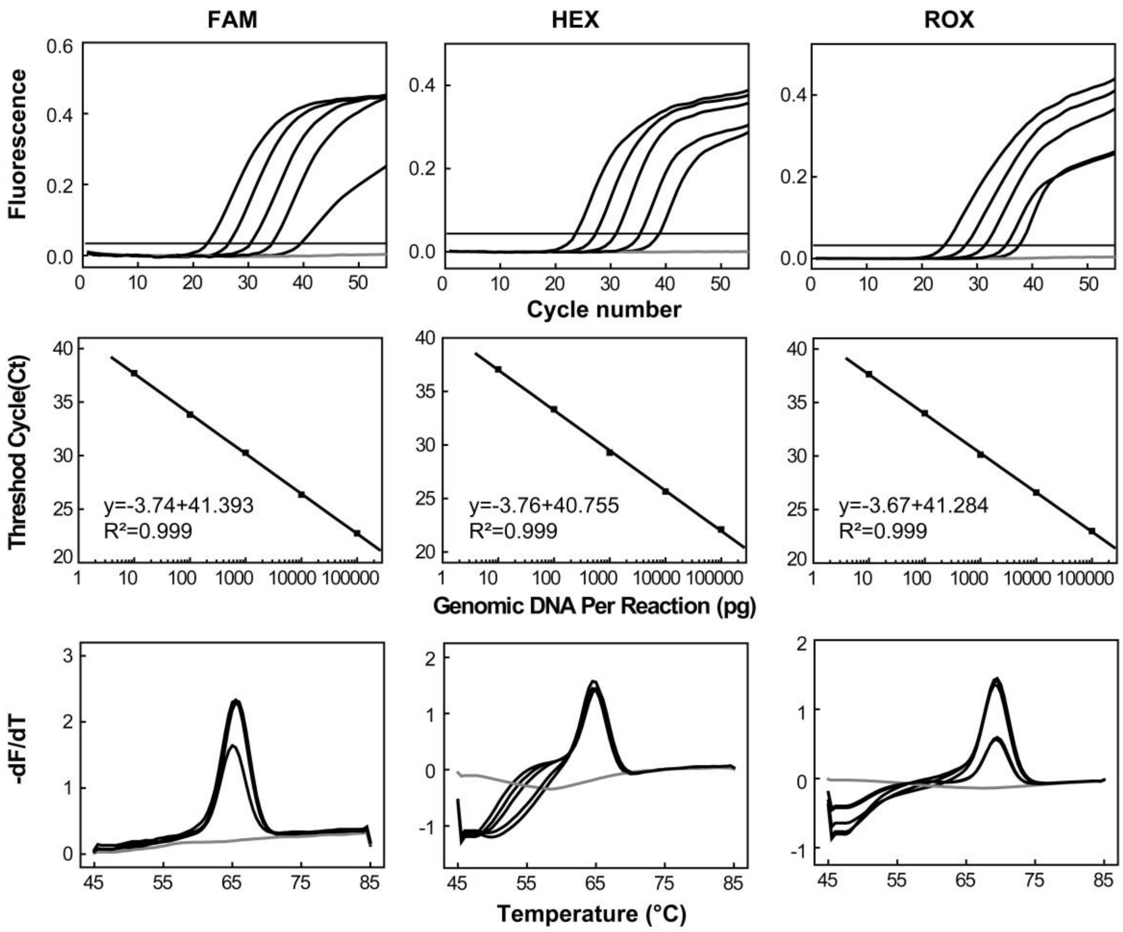

Figure S3. Analytical Sensitivity of Reaction C (*SLC26A4* and *GJB3*) by the MMCA assay. Tenfold serial dilutions of wild-type genomic DNA template ranging from 100 ng to 10 pg (from left to right) in a 25 μL PCR-reaction were performed in reaction A with three detection channels (**FAM, HEX and ROX**). **Top panel**: the PCR amplification curves in different template concentrations. **Middle panel:** Linear relationship between the number of threshold cycle values (Ct) and the logarithm of the concentration of gDNA templates. **Bottom panel:** Melting curves in different DNA templates concentration. And DNA solution was used as the no-template control (**gray lines**).

Table S1. Sequence information and concentrations of primers and probes in the MMCA assay.

	Name	Sequences (5'→3')	Concentration * (μM)	Nucleotide change	Product size (bp)
Reaction A	GJB2-F	CCGCCCAGAGTAGAAG	0.08		362
	GJB2-R	GGGTTTTGATCTCCTCGATG	0.8		
	P1	FAM-GACGATCCTGGGGGGTGTGAAC-BHQ1	0.1	c.35delG	
	P2	HEX-CAGCCAGCGTGTGCTACGATCACTAC-BHQ1	0.1	c.176_191del16	
	P3	ROX-CCGGCTATGGGCCCTGCAGCTGATCTTC-BHQ2	0.1	c.235delC	
	P4	CY5-GTGGCCTACCGGAGACGAGAAG-BHQ2	0.6	c.299_300delAT	
Reaction B	MTRNR-F	GAGTAGAGTGCTTAGTTGAACA	0.06		266
	MTRNR-R	GCGGTCAAGTTAAGTTGAA	0.6		
	MTTS-F	TCTGTAGGCTCATTCATTTCTC	0.1		265
	MTTS-R	TTGGCTTGAAACCAGCTT	1		
	P5	FAM-GAGGAGACAAGTCGTAACATGGTAAGTGT-BHQ1	0.1	m.1555A>G	
	P6	HEX-CCCGTCACCCTCCTCAAGTATAC-BHQ1	0.2	m.1494C>T	
	P7	ROX-CCCGTATACATAAAATCTAGACAAAAAAGGAAGG-BHQ2	0.1	m.7444G>A/m.7445A>G	
Reaction C	SLC26A4-F1	GGTTTAGACACAAAATCCCAGT	0.11		224
	SLC26A4-R1	GGTTGGCTCCATATGAA	1.1		
	SLC26A4-F2	TGATAGAAAAGCTGGAGCAA	0.09		162
	SLC26A4-R2	TTGACCCTCTTGAGATTTCAC	0.9		
	GJB3-F	CATCTTCAAGCTCATCATTGAGT	0.07		264
	GJB3-R	TGAGGTAGCAGAGCTCAC	0.7		
	P8	FAM-TTTGTTTTATTTCAGACGATAATTGCTACTGCC-BHQ1	0.2	c.919-2A>G	
	P9	HEX-GGTCCATGATGCTATACTCTATCTACAGAAC-BHQ1	0.1	c.2168A>G	
	P10	ROX-CTGCTACATTGCCCGACCTACCGAGAAG-BHQ2	0.1	c.538C>T/c.547G>A	

***** Final concentration of primers and probes in 25 μL PCR reaction.

Table S2. The sequences of primers used for DNA sequencing.

Gene	Primers	Sequences (5'→3')	Product size (bp)	Nucleotide change
GJB2	F1	TGGTGTTTGCTCAGGAAGA	958	c.35delG
				c.176_191del16
	R1	GCCTACAGGGGTTTCAAATG		c.235delC
				c.299_300delAT
GJB3	F2	ACTGCCTGGTACATAGTAAATG	996	c.538C>T
	R2	CCCCTGTAGGACCTCTC		c.547G>A
SLC26A4	F3	CATGTGGGAAGATTCATATGAGA	547	c.919-2A>G
	R3	TGAAGGAGTATCAGTGAAATGAAG		
SLC26A4	F4	CTGGGCAATAGAATGAGACT	266	c.2168A>G
	R4	AAGATACATCTGTAGAAAGGTTG		
MT-RNR1	F5	CCTGATGAAGGCTACAAAGTAA	517	m.1494C>T
	R5	TTCCCTTGCGGTACTATATCT		m.1555A>G
MT-TS1	F6	TCCCCTATTCTCAGGCTAC	505	m.7444G>A
	R6	CTGCATGTGCCATTAAGATAT		m.7445A>G

Table S3. Genotypes of 208 saliva gDNA samples analyzed by the MMCA assay.

Genotypes *					No. samples	
GJB2	***GJB3***	***SLC26A4***	***MT-RNR1***	***MT-TS1***	**MMCA**	**Sequencing**
c.35delG/N	WT	WT	WT	WT	1	1
c.176_191del16/N	WT	WT	WT	WT	1	1
c.176_191del16/N	WT	c.919-2A>G/N	WT	WT	1	1
c. 235delC/N	WT	WT	WT	WT	8	8
c.235delC/c.235delC	WT	WT	WT	WT	16	16
c.235delC/N	WT	c.919-2A>G/N	WT	WT	1	1
c.235delC/c.299_300delAT	WT	WT	WT	WT	2	2
c.299_300delAT/N	WT	WT	WT	WT	1	1
WT	WT	c.919-2A>G/N	WT	WT	11	11
WT	WT	c.919-2A>G/c.919-2A>G	WT	WT	8	8
WT	WT	c.2168A>G/N	WT	WT	4	4
WT	WT	c.919-2A>G/c.2168A>G	WT	WT	4	4
WT	WT	WT	m.1555A>G hom	WT	5	5
WT	WT	WT	WT	WT	145	145
Total					208	208

***** WT: wild-type; hom represents homoplasmic for the m.1555A>G mutation.
